# Editorial: Molecular and Cellular Biology of podocytes

**DOI:** 10.3389/fcell.2022.1037931

**Published:** 2022-10-10

**Authors:** Hans Kristian Lorenzo, Mario Ollero

**Affiliations:** ^1^ Faculté de Médecine, Université Paris-Saclay, Le Kremlin Bicêtre, France; ^2^ University Paris Est Creteil, INSERM, IMRB, Créteil, France

**Keywords:** podocyte, glomerulopathy, proteinuria, chronic kindey diseases, cell death, cytosqueletton

Podocytes are highly differentiated (post-mitotic) epithelial cells that are attached to the glomerular basement membrane. They are an essential part of the filtration barrier, preventing the loss of serum proteins into the urine. Because the function of podocytes constitutes the cornerstone of glomerular filtration, any alteration is likely to lead to proteinuria (nephrotic syndrome) and serious pathologies. Under normal circumstances podocytes are theoretically not replaceable and their behavior has been compared with that of neurons. Although the progressive loss of podocytes is a characteristic of healthy aging, it is particularly marked in the course of glomerular lesions (glomerulopathies). Stress response or morphological integrity are key points to establish a balance between normal and pathological states.

This Research Topic represents a snapshot of the current concerns of the nephrological community regarding this cell and its particular microenvironment. The contributions presented in this Research Topic point to several key aspects of podocyte physiology and their impact on renal function. Different points of view are considered such as 1*)* morphostructural features (cytoskeleton or slit diaphragm alterations), 2*)* mechanisms of stress response (death and loss, protection and adaptation) as well as 3*)* new methodological approaches for the study of these cells.

## 1) Structure-function relationship

Cytoskeleton integrity, always linked to morphology, is a key point in podocyte function. This is the main concern addressed by Liu et al. in their article. These authors present the WNK-OSR1/SPAK signaling pathway at the crossroads of cytoskeleton integrity, slit diaphragm function and glomerular capillary function. Another study conducted by Hamasaki et al. explores the role of dynamin 2 as a regulator of cytoskeleton function. For this purpose, the authors used a Dynamin-2 mutant (K562E) found in Charcot-Marie-Tooth disease, a sensory-motor neuropathy. This mutant revealed a reduced affinity of the cytoskeleton for the lipid membrane and led to actin disorganization in podocytes.

Another key aspect of podocyte function is the maintenance of the integrity of the slit diaphragm, a unique structure in which nephrin and beta-integrin are considered the main gatekeepers. Maywald et al. addressed the study by combining *in vivo* Drosophila experiments with *in vitro* experiments in human podocytes. The authors demonstrated that Rap1 functions downstream of nephrin signaling to β-integrin at the slit diaphragm. Additionally, a discussion on the formation and maintenance of the slit diaphragm in a Drosophila model is addressed in the review by van de Leemput et al.

## 2) Stress response mechanisms

Podocyte injury in response to stress causes their detachment from the glomerular basement membrane, followed by proteinuria and progressive renal failure. A review on the state of the art of the lesions present in podocytopathies, with a provocative interpretation of their meaning is provided by Ravaglia et al. Within the cellular and molecular characteristics of podocyte alteration, mitochondrial destabilization is present in many forms of stress associated with the pathology. In the study by S. Liu et al. alterations in podocyte mitochondria such as mitochondrial function, mitophagy, and the role of mitochondria-associated membranes are examined. Another source of stress is viral infection, such as SARS-Cov-2. Kalejaiye et al. confirm that podocytes are one of the cellular targets and characterize BSG/CD147 and ACE2 as the receptors involved in the entry of this pathogen into iPSC-derived human podocytes.

An interesting point of podocyte biology is the understanding of the mechanisms of death, protection and adaptation of these cells in response to potentially deleterious stimuli. The different causes of podocyte death, detachment and loss is a topic reviewed by Yin et al. which includes the loss of viable podocytes in the urine or the “podocyte domino effect” as a mechanism of disease progression. Other papers have addressed these points in this Research Topic. Thus, Ristov et al. show the protective effect of small molecules, such as vitamin D3 and calcipotriol. Also, Chung et al. report the beneficial effects of curcumin on podocyte viability. These authors, using a high-glucose-induced injury model, demonstrate that the reactive oxygen species (ROS) produced under these conditions are diminished after curcumin treatment. Furthermore, curcumin treatment also restored the levels of RIPK3 and other necroptosis-associated proteins, revealing an interesting protective role. Again, Liu et al. examine the protective role of calcineurin inhibitors (immunosuppressants widely used in nephrology) and demonstrated their association with WNK kinase activity. Finally, a perspective article by Lavecchia et al. explores the potential of hypertrophy, at the crossroads of cell biology and biophysics, as an adaptive response to podocyte injury.

## 3) Experimental and methodological issues

Another key aspect of research on glomerulopathies is the need for robust experimental models (especially *in vitro*), so the study and characterization of the ones available and the development of new ones is crucial. Some of the articles in this Research Topic address this topic. Thus, thanks to the study by Bryant et al. we know that the administration of adriamycin, a widely accepted model of nephropathy, induces proteinuria depending on the genetic background of mice. In this study, the authors analyze in depth the impact of the C57B6 background, usually considered as resistant, and show the differences between two substrains, providing valuable information for *in vivo* assays. One of the major methodological problems consists of having *in vivo* access to podocytes in mammals. The use of Drosophila nephrocytes has recently been shown as an alternative model due to their similarity at the genetic, molecular and functional level to mammal podocytes. The challenges associated with *in vitro* study of the slit diaphragm using Drosophila nephrocytes is reviewed by van de Leemput et al. Another methodological problem is the weakness of podocytes in culture as a model to study new drugs and te difficulties to translate the results to the clinical level. This is due to the complex structure of the glomerular filtration barrier, which is barely represented by a simple podocyte culture. To overcome this problem, Ristov et al. propose an innovative assay to examine glomerular function. This is the so-called “GlomAssay”, a semi-automated high-throughput screening method that allows the study of hundreds of compounds in combination with pathway analysis (transcriptomics and proteomics). Its robustness is demonstrated within the study on the protective role of vitamin D3 in podocytes discussed above.

